# ANK1 and DnaK-TPR, Two Tetratricopeptide Repeat-Containing Proteins Primarily Expressed in *Toxoplasma* Bradyzoites, Do Not Contribute to Bradyzoite Differentiation

**DOI:** 10.3389/fmicb.2017.02210

**Published:** 2017-11-13

**Authors:** Jichao Yang, Lihong Zhang, Huiyan Diao, Ningbo Xia, Yanqin Zhou, Junlong Zhao, Bang Shen

**Affiliations:** ^1^State Key Laboratory of Agricultural Microbiology, Huazhong Agricultural University, Wuhan, China; ^2^Hubei Cooperative Innovation Center for Sustainable Pig Production, Wuhan, China; ^3^Key Laboratory of Preventive Medicine in Hubei Province, Wuhan, China

**Keywords:** *Toxoplasma gondii*, bradyzoite, ANK1, DnaK-TPR, stage conversion

## Abstract

*Toxoplasma gondii* is an important zoonotic pathogen infecting one third of the world population and numerous animals. A key factor to its wide distribution is the ability to interconvert between fast replicating tachyzoites and slowly growing bradyzoites, and to establish lifelong chronic infection in intermediate hosts. Although it is well accepted that stage conversion plays key roles in the pathogenesis and transmission of the parasite, little is known about the molecular mechanisms behind it. Using existing gene expression data from TOXODB and published work, we looked for proteins with novel functional domains and whose expression is up-regulated in the bradyzoite stage, hoping to find molecules that have critical roles in regulating stage conversion and bradyzoite formation. In this study we characterized two such proteins ANK1 and DnaK-TPR, both of which are primarily expressed in bradyzoites and contain novel motifs to mediate protein-protein interactions. Through CRISPR/CAS9 directed gene editing technology, both genes were individually knocked out in type 1 strain TgHB2 and type 2 strain ME49. Disruption of neither of these two genes affected the growth or replication of tachyzoites *in vitro*, consistent with their minimal expression at this stage. However, mutants lacking *ANK1* or *DnaK-TPR* displayed modest virulence attenuation during mice infection. Surprisingly, inactivation of neither *ANK1* nor *DnaK-TPR* seemed to have a significant impact on bradyzoite differentiation *in vitro* or cyst formation *in vivo*. These results suggest that ANK1 and DnaK-TPR probably do not directly contribute to bradyzoite differentiation, but likely affect other aspects of bradyzoite biology.

## Introduction

*Toxoplasma gondii* is an obligate intracellular parasite belonging to the phylum apicomplexa. It is capable of infecting virtually all warm-blooded animals and humans, and has a world-wide distribution. It is estimated that 30% of the world population is infected by this parasite, causing congenital toxoplasmosis and other severe complications in susceptible individuals and animals, which leads to great economical losses and public health problems (Elmore et al., 2010; Dubey et al., 2012). *T. gondii* has a complex life cycle that consists of two phases: the sexual phase, which takes place in felines such as domestic cats, and the asexual phase, which takes place in all warm-blooded animals and humans (Dubey, 1998; Dabritz and Conrad, 2010). In intermediate hosts such as humans, *T. gondii* can exist in two forms: a fast replicating form called tachyzoite and a slowly growing form called bradyzoite. During the infection of a naive host, *T. gondii* first quickly multiplies as tachyzoites to spread the parasites and establish systemic infection. Therefore tachyzoite is responsible for acute toxoplasmosis. Nonetheless, *T. gondii* infection rarely causes obvious symptoms in healthy people. The reason for that is, once the immune responses against *T. gondii* infection are activated, tachyzoites are effectively eliminated. As a response to host immune clearance, some of the tachyzoites convert to the slowly replicating bradyzoites and stay within host cells (mainly neurons and muscle cells) in the form of tissue cysts, to establish chronic infection and escape immune surveillance (Remington and Merigan, 1968; Dubey et al., 1998; Weiss and Kim, 2000). Once formed, chronic infection will stay with the hosts lifelong, and may get reactivated when hosts’ immune function is compromised (Shen et al., 2016). Currently there are no effective treatments for chronic toxoplasmosis.

In addition to hosts’ immune pressure, bradyzoites can also be induced *in vitro* by a variety of conditions (Bohne et al., 1993; Soete et al., 1994; Weiss et al., 1995). In general, it is thought that bradyzoite differentiation is a stress response parasites initiate to adapt to adverse environmental conditions (Ferreira da Silva Mda et al., 2008). Nutrient starvation, extreme pH and respiratory chain inhibitors such as antimycin A are all able to induce bradyzoite formation *in vitro* (Soete et al., 1994). Alkaline medium with pH 8.2 along with CO_2_ starvation (ambient CO_2_) is the most common way of inducing bradyzoite *in vitro* (Weiss et al., 1995). Although the exact mechanisms of how parasites sense the stress are currently unknown, it is clear that stress conditions induce global changes in gene expression that eventually lead to slow-down of cell cycle and formation of bradyzoites (Manger et al., 1998; Skariah et al., 2010; Buchholz et al., 2011).

The interconversion between tachyzoites and bradyzoites plays a critical role in the pathogenesis and transmission of *T. gondii*. In order to understand the biology and mechanisms underlying such stage conversion, micro-array and RNAseq analysis have been done to compare gene expression differences between tachyzoites and bradyzoites, and hundreds of genes were reported to be differentially regulated (Buchholz et al., 2011; Pittman et al., 2014). These differentially regulated genes were thought to play important roles during stage conversion. Indeed, some of the up-regulated genes were experimentally shown to be important for bradyzoite differentiation and cyst formation, such as the bradyzoite specific glycolytic enzyme ENO1 (Mouveaux et al., 2014) and the cyst wall protein CST1 (Tomita et al., 2013).

In an effort to understand the molecular basis for *T. gondii* stage conversion, we examined the differentially expressed proteins between tachyzoites and bradyzoites, and looked for proteins that have novel functional motifs. In this study, we characterized two proteins that contain tetratricopeptide repeat (TPR) structural motifs. Originally identified in yeast (Hirano et al., 1990; Sikorski et al., 1990), TPR motifs are ubiquitously found in a variety of proteins from bacteria to humans (Allan and Ratajczak, 2011). As the name indicates, TPR motifs consist of regions that are 34-residues long (Cerveny et al., 2013). They are minimally conserved sequence wise, but consensus patterns of small and large hydrophobic residues combination have been defined (Cerveny et al., 2013). Structural analysis on the TPR motifs of protein phosphatase 5 (PP5) and many other TPR containing proteins indicate that they form two antiparallel α-helices that are equivalent in length (Das et al., 1998; Allan and Ratajczak, 2011). The basic function of TPR motifs is to form structural scaffold to mediate protein-protein interactions. TPR motifs are present in many proteins that are involved in different cellular processes and functions, such as gene expression regulation, protein transport, bacterial virulence, and immune functions (Allan and Ratajczak, 2011; Cerveny et al., 2013). Based on bioinformatic prediction, *T. gondii* genome encodes more than 50 TPR containing proteins. Of these, *ANK1* (TGME49_216140) and *DnaK-TPR* (TGME49_202020) are primarily expressed at the bradyzoite stage (Friesen et al., 2008; Ueno et al., 2011). In this study we used gene knockout strategies to analyze their roles during *T. gondii* growth and development.

## Materials and Methods

### Cells and Parasites

Tachyzoites of *T. gondii* strains ME49 and TgHB2 were propagated in human foreskin fibroblasts (HFF), which were cultured in Dulbecco’s modified Eagle medium (DMEM) supplemented with 10% fetal bovine serum (FBS), 100 μg/ml streptomycin and 10 mM L-glutamine. The genetically modified parasites were propagated and cultured under the same conditions.

### Searching for Differentially Regulated Proteins with Novel Function Motifs

Gene expression datasets available from ToxoDB were used for this purpose. First, the “Oocyst, tachyzoite, and bradyzoite developmental expression profiles (M4)” dataset was searched for protein coding genes whose expression had a fold change greater than 10 between the tachyzoite (2 days *in vitro*) and bradyzoite (8 days *in vitro*) stages. Second, the “Transcriptome during acute or chronic infection in mouse brain” dataset was searched using the same parameters to find genes that had 10-fold difference between “acute infection 10 days p.i.” and “chronic infection 28 days p.i.”. Third, the “Bradyzoite Differentiation Expression Profiles (ME49, GT1, CTGara)” dataset was used to find genes that had 10-fold expression difference between “ME49 tachyzoite” and “ME49 pH 8.2.” Subsequently, genes that were identified in all three searches were collected and analyzed manually. There were 18 genes identified in total, which included lactate dehydrogenase *LDH2*, bradyzoite antigen *BAG1*, bradyzoite rhoptry protein *BRP1* and other well described bradyzoite specific genes. Judging from the “Product Production” provided by ToxoDB, two (ANK1 with the gene ID TgME49_216140 and DnaK-TPR with the gene ID TgME49_202020) out of the 18 proteins contained tetratricopeptide repeats (TPR), which then became the focus of this study.

To further characterize the TPR containing proteins, their primary sequences were used in protein Blast searches^1^, as well as in the Motif Scan^2^ server to search the Prosite and Pfam databases, to identify potential function domains.

To predict the number and positions of the TPR motifs in TPR containing proteins, their sequences were analyzed by the profile based method TPRpred^3^ (Karpenahalli et al., 2007), using the *P*-value threshold of 10^-4^.

### Deletion of *ANK1* or *DnaK-TPR* Genes Using the CRISPR/CAS9 System

To construct homologous templates for *ANK1* or *DnaK-TPR* knockouts, the 5′- and 3′- homology arms of the corresponding genes were cloned into the pUC19 vector along with the selection marker *DHFR*, using multi-fragment cloning. The primers used to amplify the homology arms from genomic DNA of ME49 were listed in **Table 1**. After obtaining each fragment, 5′ homology arm, *DHFR* and 3′ homology arm were ligated into the linearized pUC19 (achieved through SacI and HindIII digestion) vector by the One Step Cloning Kit (ClonExpress II One Step Cloning Kit, Vazyme, United States) according to manufacturer’s instructions. The gene specific CRISPR plasmids were constructed by site directed mutagenesis of the *UPRT* targeting CRISPR plasmid, as previously described (Shen et al., 2014, 2017). Primers used for mutagenesis were also listed in **Table 1**. All constructs were confirmed by Sanger sequencing before use.

**Table 1:** Primers used in this study.

Name	Sequence	Used for
gRNA-216140-Fw	GCGCTGTGAGATGAATGCGTGTTTTAGAGCTAGAAATAGC	*ANK1* specific CRISPR plasmid construction
gRNA-CRISPR-Rv	AACTTGACATCCCCATTTAC	Gene specific CRISPR plasmid construction
gRNA-202020-Fw	CAAGCTCAGGGCATTCCTGGGTTTTAGAGCTAGAAATAGC	*DnaK-TPR* specific CRISPR plasmid construction
5H-2020-Fw	ACGACGGCCAGTGAATTCGAGCTCATCCCGCAACAGATGTGTCG	Amplification of 5′ homology arm of *DnaK-TPR* for multi-fragment cloning
5H-2020-Rv	CTCACGGGATTTACAGCCTGAGTGTCCGCAGATCAACTGG	
DHFR-Fw	CAGGCTGTAAATCCCGTGAG	Amplification of *DHFR* from pUPRT::DHFR-D for multi-fragment cloning
DHFR-Rv	GATTCCGTCAGCGGTCTGTC	
3H-2020-Fw	TGACAGACCGCTGACGGAATCAGCTTGAAACAGCGTCGG	Amplification of 3′ homology arm of *DnaK-TPR* for multi-fragment cloning
3H-2020-Rv	GCTATGACCATGATTACGCCAAGCTTTCAGCCTCTCGAATAACCTC	
5′-Up2020-Fw	AACCGCATGGATGGCTACCG	PCR1 for Δ*dnaK-tpr* mutants
3′-InDHFR-Rv	GACAGGACGCTACTGGGACTG	PCR1 for Δ*dnaK-tpr* and Δ*ank1* mutants
5′-InDHFR-Fw	CACGACAGCAGACAACTTTC	PCR2 for Δ*dnaK-tpr* and Δ*ank1* mutants
3′-Dn2020-Rv	CCCGATCCATTCGTTCCAGC	PCR2 for Δ*dnaK-tpr* mutants
5′-In2020-Fw	TTGGCGCATGAGTAAAGCGG	PCR3 for Δ*dnaK-tpr* mutants
3′-In2020-Rv	CTTCGTGACTTACGAGGAGC	
5H-ANK1-Fw	ACGACGGCCAGTGAATTCGAGCTCGACGGCGTACGTTATCGATG	Amplification of 5′ homology arm of *ANK1* for multi-fragment cloning
5H- ANK1-Rv	CTCACGGGATTTACAGCCTGAGGTTACCGTACGCCAAGC	
3H- ANK1-Fw	TGACAGACCGCTGACGGAATCATGAACGTACGCGTGCAAC	Amplification of 3′ homology arm of *ANK1* for multi-fragment cloning
3H- ANK1-Rv	GCTATGACCATGATTACGCCAAGCTTGTCTGTGATCTGTCTCGGGC	
5′-Up ANK1-Fw	CCTCTACGCACTTGAAACACCC	PCR1 for Δ*ank1* mutants
3′-Dn ANK1-Rv	CTGTTGGCTGAATGTTCCACG	PCR2 for Δ*ank1* mutants
5′-In ANK1-Fw	CCGTGTGGGACAGATATTAC	PCR3 for Δ*ank1* mutants
3′-In ANK1-Rv	TGCAATGCAATCCTGACGTG	
DnaK-TPR-Fw	CACAGACAACAGAAGCCGTT	RT-PCR for *DnaK-TPR*
DnaK-TPR-Rv	TCGTTGGAATAGAGCGTCTG	
ANK1-Fw	GCGAAGAATCAAAGTGACGA	RT-PCR for *ANK1*
ANK1-Rv	GCCTCGACATCGTTATAGCA	
GAPDH-Fw	GGTGTTCCGTGCTGCGAT	RT-PCR for *GAPDH*
GAPDH-Rv	GCCTTTCCGCCGACAAT	

To generate gene knockouts in desired strains, corresponding homology construct and gene specific CRISPR plasmid were co-transfected into freshly egressed tachyzoites, as described previously (Shen et al., 2017). Subsequently transfectants were selected with 1 μM pyrimethamine for 3 – 4 passages until the drug resistant pools became stable. Single clones were isolated through limiting dilution in 96-well plates and examined by diagnostic PCRs (PCR1, PCR2 and PCR3; primers are listed in **Table 1**) to check the disruption of corresponding genes.

### Quantitative Real-Time PCR

Approximately 1 × 10^7^ freshly egressed parasites (tachyzoites or bradyzoites) of the ME49 strain were collected and purified through polycarbonate membranes with the pore size of 3 μm. Parasites were washed with PBS and total RNA was extracted from them using the trizol methods, according to manufacturer’s instructions (TransGen Biotech, China). Bradyzoites were obtained by growing parasites in differentiation medium (RPMI 1640 medium supplemented with 1% FBS and 50 mM HEPES, pH 8.2) along with ambient CO_2_ for 4 days (Walker et al., 2013). After RNA isolation, 2 μg total RNA from each sample was reverse transcribed into cDNA using the PrimeScript RT reagent Kit (Takara Bio, Japan).

Subsequently the cDNA was used as template for real-time PCR (RT-PCR) to estimate the expression levels of target genes. Three pairs of specific primers were designed for this purpose: ANK1-Fw and ANK1-Rv; DnaK-TPR-Fw and DnaK-TPR-Rv; GAPDH-Fw and GAPDH-Rv (Sequences are listed in **Table 1**). RT-PCR was performed on the ViiA-7 Real-Time system (Life Technologies, Camarillo, CA, United States) using the SYBR Green method, according to the manufacturer’s instructions. Cycling conditions for RT-PCR were set as: initial denaturation at 95°C for 30 s, followed by 40 cycles of 95°C for 15 s, 56°C for 30 s and 72°C for 20 s. Each sample was prepared and examined three times independently. Gene expression levels was estimated by the Δ^*C*_T_^ method using the *GAPDH* gene as internal reference, as described previously (Selseleh et al., 2012).

### Whole Genome Sequencing

Freshly egressed tachyzoites (2 × 10^7^) were purified through membrane filtration and washed with PBS. Subsequently genomic DNA was extracted from the purified parasites using the EasyPure Genomic DNA Kit (Transgen Biotech, China). Purified genomic DNA was sheared into short fragments of about 350 bps by sonication and then used for library construction using the TruSeq Library Construction Kit (Illumina, San Diego, CA, United States) according to the manufacturer’s instructions. The libraries were then sequenced by the Illumina HiSeq platform (Illumina, San Diego, CA, United States). The average sequencing depth was around 110X. To detect genetic alterations, clean reads obtained from genome sequencing were mapped to the reference genome of GT1, and SAMTOOLs, BreakDancer and CNVnator were used to detect short indels, structural variations (insertion, deletion, and inversion) and copy number variations, respectively (Chen et al., 2009; Li et al., 2009; Abyzov et al., 2011). The mapping results were visualized by the Integrative Genomics Viewer (Thorvaldsdottir et al., 2013).

### Plaque Assay

Human foreskin fibroblasts cells were seeded into six-well plates and grown at 37°C until confluent. Subsequently 100 purified tachyzoites were added to each well and cultured for 10–12 days without disturbance under standard growth conditions. Then the samples were fixed with 4% paraformaldehyde and stained with 0.5% crystal violet to develop the plaques. Finally the plates were scanned by a scanner (FileScan 380, Microtek, China) and the number and sizes of plaques in each well were determined as described previously (Shen and Sibley, 2014). Each strain was tested three times independently, each with triplicates.

### Intracellular Replication Assay

Coverslips in 24-well plates seeded with confluent HFF monolayers were infected with freshly harvested tachyzoites (10^5^ parasites per well) for 1 h at 37°C. Extracellular parasites that failed to invade HFF cells were washed away and invaded ones were grown for another 24 h under standard tachyzoite growth conditions. Subsequently the samples were fixed with 4% paraformaldehyde and stained with swine anti-Toxoplasma (gifts from the Zhao lab at Huazhong Agricultural University, to stain extracellular parasites). After extensive washing, samples were permeabilized with 0.1% Triton X-100 for 10 min and stained with rabbit anti-TgALD (gifts from the Sibley lab at Washington University in St. Louis, to stain all parasites). Primary antibodies were detected by Alexa Fluor 594-conjugated goat anti-rabbit IgG and FITC conjugated goat anti-swine IgG secondary antibodies, respectively (Life Technologies, Camarillo, CA, United States). Parasites that were stained red but not green (these were intracellular parasites) were analyzed to determine the number of parasites in each parasitophorous vacuole (PV). Each sample was tested three times independently, each with triplicates.

### Bradyzoite Differentiation Assay

Alkaline medium was used to induce bradyzoite differentiation *in vitro*, as described previously (Walker et al., 2013). Briefly, Purified parasites were used to invade HFF monolayer (10^4^ parasites/well) seeded on coverslips in 24-well plates for 1 h under standard growth conditions. Subsequently non-invaded parasites were washed away with PBS and the rest of the cultures were grown in differentiation medium with ambient CO_2_ for 4 days. After treatment, samples were fixed with 4% paraformaldehyde, permeablized with 0.1% Triton X-100, and stained with rabbit anti-TgALD and mouse anti-TgBAG1 (gifts from the Zhao lab at Huazhong Agricultural University) antibodies. Alexa Fluor 594-conjugated goat anti-rabbit IgG and Alexa Fluor 488-conjugated goat anti-mouse IgG secondary antibodies (Life Technologies, Camarillo, CA, United States) were used to detect primary antibodies. Hoechst 33342 was used to stain cell nuclei. Parasites that were stained both green and red were counted as bradyzoites, whereas parasites that were stained only red were treated as tachyzoites. Bradyzoite differentiation efficiency was determined by dividing the number of green vacuoles by the number of red vacuoles. At least 100 total vacuoles were counted for each sample in each experiment and each sample was repeated three times independently.

### Animal Experiments

All 4–6 weeks old female ICR mice and 10–12 weeks old Kunming mice were purchased from the Hubei Provincial Centers for Disease Control. To test the virulence of parasites in mice, freshly harvested tachyzoites were purified by filtration through 3 μm polycarbonate membranes and then used to infect mice by intraperitoneal injection. Each parasite strain was tested by infection of 10 mice at the dose of 100 tachyzoites per mouse. After infection, mice were monitored daily for 30 days. The cumulative mortality was calculated by dividing the number of mice that died by the number of mice infected. At the conclusion of the virulence tests, mice that survived the infection at day 30 were sacrificed and brain tissues were homogenized to determine the number of *Toxoplasma* cysts by DBA-FITC staining, as described previously (Buchholz et al., 2013). All animal experiments were approved by The Scientific Ethic Committee of Huazhong Agricultural University (permit #: HZAUMO-2016-041).

## Results

### Sequence Analysis of ANK1 and DnaK-TPR Proteins

**FIGURE 1:**
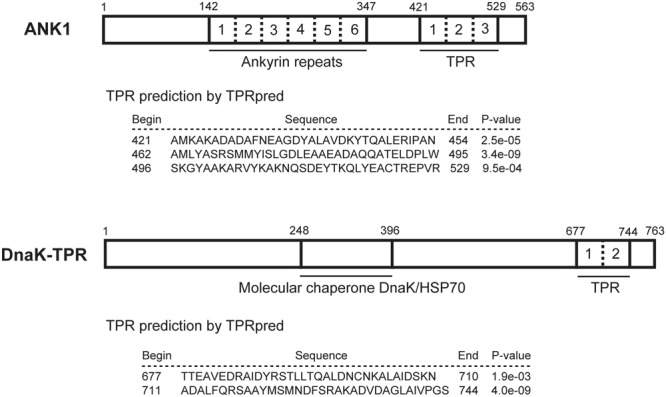
Functional domains within ANK1 and DnaK-TPR proteins. The amino acid sequences of ANK1 and DnaK-TPR were subject to BLAST search, as well as motif scanning against Pfam and PROSITE databases. TPR domains were predicted by the online tool TPRpred. Numbers above the boxes indicate the amino acid positions. Different ankyrin repeats or TPR motifs within a protein were separated by dashed lines and individually numbered.

Both ANK1 and DnaK-TPR have been reported previously as proteins whose expression was drastically increased during tachyzoite to bradyzoite conversion. *ANK1* was initially reported as 35.m00032 and *DnaK-TPR* was reported as 20.m00351 (Friesen et al., 2008). According to the current gene model on ToxoDB, ANK1 is a multi-domain protein with 563 amino acids. Sequence analysis using BLAST, Pfam and PROSITE database searching indicated that it has an ankyrin repeat domain in the middle with 5 – 7 ankyrin repeats next to each other, depending on the program used (**Figure 1**). The TPR prediction program TPRpred (Karpenahalli et al., 2007) predicted that it likely has 3 TPR motifs near the C-terminus. The positions and sequences of these TPR motifs were shown in **Figure 1**. DnaK-TPR is also a multi-domain protein with 763 amino acids. Sequence analysis suggested that it has a DnaK/HSP70 family domain with molecular chaperone function in the central part (roughly between residues 248 and 396). TPRpred predicted that it may have two TPR motifs near the end of the protein, the one between A711 and S744 is of high probability (**Figure 1**).

### Differential Expression of *ANK1* and *DnaK-TPR* at the Tachyzoite and Bradyzoite Stages

**FIGURE 2:**
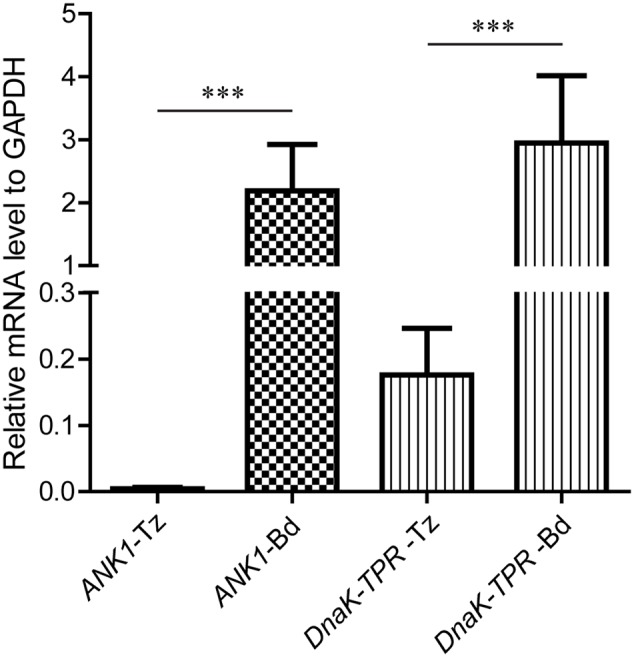
Relative expression levels of *ANK1* and *DnaK-TPR* in *Toxoplasma* tachyzoites and bradyzoites. Total RNA was extracted from 10^7^ tachyzoites or alkaline induced bradyzoites of ME49 and reverse transcribed into cDNA. Transcript levels for *ANK1* and *DnaK-TPR* in each sample were estimated by quantitative real-time PCR, using *GAPDH* as an internal reference. Means ± SD of three independent experiments. ^∗∗∗^*P* < 0.0001, Student’s *t*-test. Tz, tachyzoites; Bd, bradyzoites.

Previous studies (Friesen et al., 2008; Ueno et al., 2011), as well as gene expressing data (Micro-array and RNA-Seq) from ToxoDB, all suggested that the expression of *ANK1* and *DnaK-TPR* was significantly different between the tachyzoite and bradyzoite stages. To quantitatively analyze the expression differences of these two genes between the tachyzoite and bradyzoite stages, real-time PCR (RT-PCR) was performed, using cDNA reverse transcribed from total RNA of tachyzoites or *in vitro* induced bradyzoites of the ME49 strain. Bradyzoites were obtained by growing parasites in differentiation medium for 4 days with ambient CO_2_. Using *GAPDH* expression levels as reference, RT-PCR results showed that expression of *ANK1* was barely detectable at the tachyzoite stage, but increased almost 500-fold when cultured under bradyzoite inducing conditions (**Figure 2**). Similarly, there was a 16-fold increase in the transcript level of *DnaK-TPR* upon bradyzoite induction (**Figure 2**). Therefore, expressions of both *ANK1* and *DnaK-TPR* were indeed significantly up-regulated upon bradyzoite induction, consistent with the data from ToxoDB and previous studies (Friesen et al., 2008; Ueno et al., 2011). In addition, using alkaline media or CO_2_ starvation to treat the parasites, the Roos lab at University of Pennsylvania looked at gene expression changes every 6 h upon bradyzoite induction. Their data deposited in ToxoDB suggest that obvious increase in *DnaK-TPR* expression was observed 36 h post either treatment. Whereas for *ANK1*, rise in expression was seen as early as 12 h upon alkaline stress, but did not become apparent until 36 h after CO_2_ starvation. These results imply that both *ANK1* and *DnaK-TPR* are early induced genes during bradyzoite conversion.

### Generation of *ANK1* or *DnaK-TPR* Knockout Strains Using the CRISPR/CAS9 System

**FIGURE 3:**
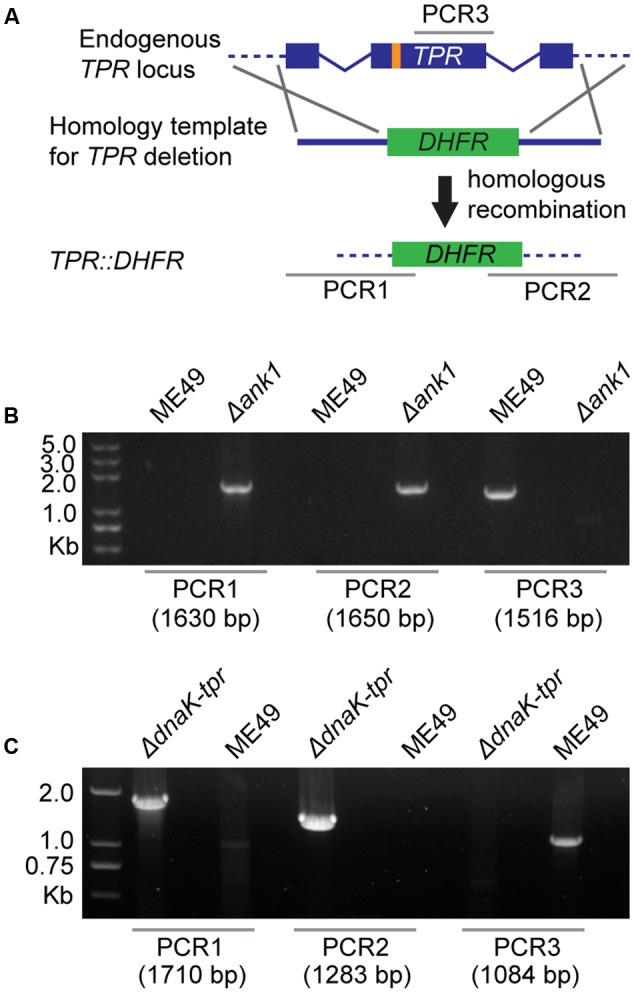
Deletion of *ANK1* and *DnaK-TPR* using the CRISPR/CAS9 technology. **(A)** Schematic illustration of knocking out *ANK1* or *DnaK-TPR* genes (*TPR)* by CRISPR/CAS9 directed homologous gene replacements. Orange bar indicates the CRISPR targeting site, whereas PCR1/2/3 denote the diagnostic PCRs for knockout strain identification used in **(B,C)**. PCR1 and PCR2 check the 5′ and 3′ integration of the pyrimethamine resistant marker *DHFR*, whereas PCR3 examines the deletion of target genes. **(B)** Diagnostic PCRs on a ME49 Δ*ank1* clone. **(C)** Diagnostic PCRs on a ME49 Δ*dnaK-tpr* clone.

**FIGURE 4:**
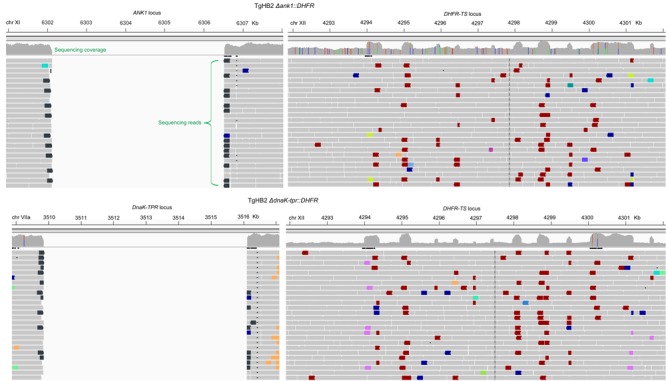
Confirm gene deletions by whole genome sequencing. Genomic DNA was extracted from tachyzoites of TgHB2 Δ*ank1* or TgHB2 Δ*dnaK-tpr* and subject to genome sequencing. Subsequently the clean reads were mapped to the reference genome of the GT1 strain and visualized by the Integrative Genomics Viewer. The numbers above each image indicate the chromosomal position of the corresponding gene in GT1.

To study the functions of ANK1 and DnaK-TPR during *T. gondii* growth and development, the CRISPR/CAS9 genome editing technique was used to delete these genes in type 2 strain ME49 and type 1 strain TgHB2. The strategy for deleting target genes by CRISPR/CAS9 mediated homologous recombination was illustrated in **Figure 3A**. After transfecting the gene specific CRISPR plasmid and homology template into corresponding parental strain (ME49 or TgHB2), transfectants were selected with pyrimethamine and single cloned by limiting dilution. Single clones were then screened by diagnostic PCR to check the correct replacement of target genes by the selection marker *DHFR* (PCR1/2/3 in **Figure 3A**). Using this strategy, *ANK1* and *DnaK-TPR* single deletion mutants were generated in both ME49 and TgHB2. Diagnostic PCR results for one clone of ME49 Δ*ank1* and one clone of ME49 *ΔdnaK-tpr* were shown in **Figures 3B,C**, respectively. Results for the same mutants produced in TgHB2 were identical to that made in ME49, therefore were not shown here. To further confirm the successful knockout of these genes, we performed whole genome sequencing on the two mutants made in the TgHB2 background. For both TgHB2 Δ*ank1* and TgHB2 Δ*dnaK-tpr*, complete absence of target gene was observed (**Figure 4**). Meanwhile, we detected increased sequencing coverage for the exons of the *DHFR-TS* gene, which corresponds to the increased copy number of *DHFR* as a consequence of replacing target genes with *DHFR* (**Figure 4**). Together, these results confirmed the complete deletion of the *ANK1* and *DnaK-TPR* genes, successful disruption of these genes indicated that neither gene is required for tachyzoite survival, which is consistent with their low expression at the tachyzoite stage.

### Impact of ANK1 and DnaK-TPR on Tachyzoite Growth *in Vitro*

**FIGURE 5:**
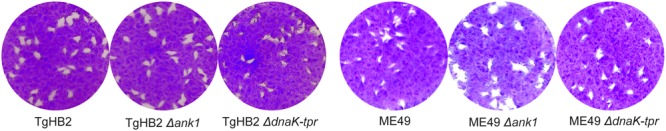
Growth of *ANK1* or *DnaK-TPR* knockouts as tachyzoites *in vitro* determined by plaque assay. HFF monolayers infected with 100 tachyzoites of indicated strains were cultured for 10 or 12 days at 37°C, fixed with 70% ethanol, and then stained with crystal violet to develop the plaques.

After obtaining the knockout mutants, we sought to determine the contribution of these genes to the growth of *T. gondii* tachyzoites in detail. First, plaque assay was used to assess the overall growth rates of these mutants. As shown in **Figure 5**, after 10 or 12 days’ growth, both *ANK1* and *DnaK-TPR* deletion mutants formed similar number and sizes of plaques on HFF host cell monolayer as the parental strain did. Mutants made in both ME49 and TgHB2 produced plaques with similar efficiencies as their corresponding parental strain. These results suggested that neither ANK1 nor DnaK-TPR had a significant impact on tachyzoite growth *in vitro*.

**FIGURE 6:**
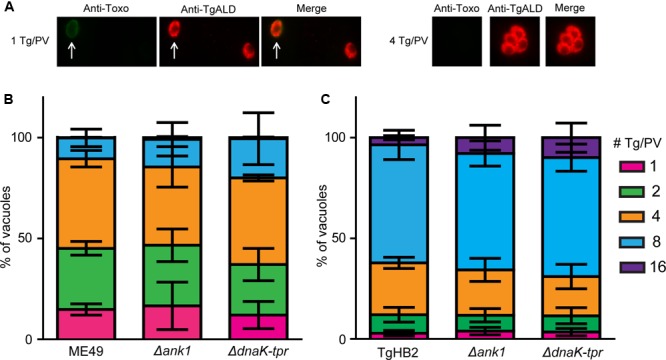
Intracellular replication of *ANK1* and *DnaK-TPR* single deletion mutants *in vitro*. Freshly egressed tachyzoites purified from indicated strains were used to infect HFF monolayer and invaded parasites were cultured for 24 h before fluorescent staining to determine the number of parasites in each parasitophorous vacuole (PV). Swine anti-Toxoplasma (Anti-Toxo) was used to stain extracellular parasites, which were excluded from the intracellular replication analysis. Rabbit anti-TgALD was used to stain total parasites. **(A)** Examples of PV containing 1 or 4 tachyzoites. The white arrow indicated an extracellular parasite, such parasites were not included in analysis. **(B,C)** Percentiles of PV containing indicated number of parasites for wild type and mutant strains. Means ± SD of three independent experiments, each with triplicates.

To further check the parasite growth *in vitro*, intracellular replication assay was performed to check the proliferation efficiency. Tachyzoites of each strain were used to infect HFF cells for 1 h and successfully invaded parasites were allowed to replicate for 24 h under standard growth conditions. Then the number of parasites in each parasitophorous vacuole (PV) was determined by immunofluorescent staining of *Toxoplasma* aldolase (**Figure 6A**). The results showed that both *ANK1* or *DnaK-TPR* deletion mutants displayed very similar replication dynamics as the parental strains (**Figures 6B,C**), suggesting that ANK1 and DnaK-TPR do not play critical roles for tachyzoite replication *in vitro*.

### Neither *ANK1* nor *DnaK-TPR* Inactivation Affected Bradyzoite Formation *in Vitro*

**FIGURE 7:**
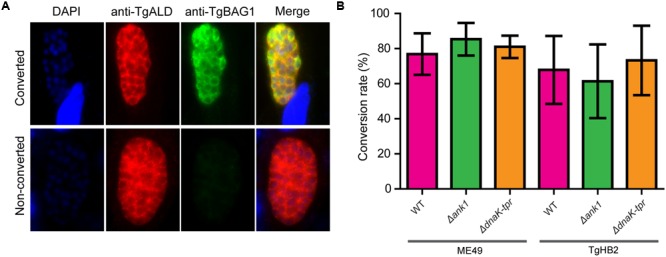
Bradyzoite differentiation efficiency of the *ANK1* or *DnaK-TPR* knockout mutants *in vitro*. HFF monolayers infected with indicated parasite strains were cultured under alkaline conditions (pH 8.2, ambient CO_2_) for 4 days. Samples were then fixed and stained with mouse anti-BAG1 (a bradyzoite specific marker) and rabbit anti-TgALD (stain of total parasites), to calculate the efficiency of bradyzoite differentiation (# of BAG1^+^ vacuoles/# of ALD^+^ vacuoles). **(A)** Examples of parasites that were converted (BAG1^+^) or not converted (BAG1^-^) to bradyzoites. **(B)** Bradyzoite conversion rates of indicated strains, mean ± SD of three independent experiments.

As genes whose expressions were significantly up-regulated at the bradyzoite stage, they were hypothesized to play roles in bradyzoite differentiation or development. To check these possibilities, mutant strains were induced with alkaline conditions to form bradyzoites *in vitro*, which were detected by positive staining of a bradyzoite marker BAG1 (**Figure 7A**). After growing parasites under the differentiation condition for 4 days, about 80% of ME49 PV turned BAG1 positive, indicating a high efficiency of bradyzoite transition (**Figure 7B**). ME49 Δ*ank1* and ME49 Δ*dnaK-tpr* displayed very similar transition rates as the parental strain ME49. Under this condition, about 70% of the TgHB2 PV became BAG1 positive (**Figure 7B**), which is slightly lower than that of ME49, consistent with the type 1 nature of this strain. Nonetheless, *ANK1* or *DnaK-TPR* deletion mutants made in this strain also had similar bradyzoite formation efficiencies as the parental strain, suggesting that indeed these two genes did not have significant roles in bradyzoite formation *in vitro*.

### Both *ANK1* and *DnaK-TPR* Single Deletions Slightly Attenuated Parasite Virulence in Mice

**FIGURE 8:**
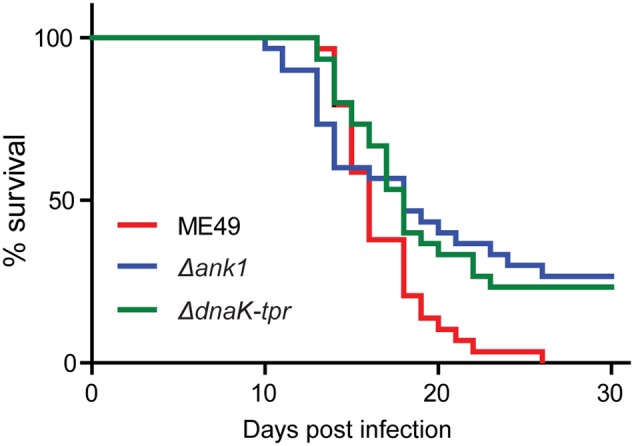
Acute virulence of the *ANK1* and *DnaK-TPR* single deletion mutants in mice. ICR mice were infected with freshly egressed tachyzoites (100 parasites/mouse, 40 mice for each strain) of indicated strains by intraperitoneal injection. Subsequently their survival was monitored daily for 30 days.

To check the effect of *ANK1* or *DnaK-TPR* disruption on parasite virulence *in vivo*, tachyzoites of mutant strains were used to infect ICR mice and the survival of mice were monitored. The results for the ME49 serials of strains (ME49, ME49 Δ*ank1*, Me49 Δ*dnaK-tpr*) were shown in **Figure 8**. At the infection dose of 100 tachyzoites per mouse, the parental strain ME49 killed all the mice, whereas both *ANK1* and *DnaK-TPR* single deletion mutants killed about 70% of infected mice, suggesting that the virulence of both *ANK1* and *DnaK-TPR* mutants was slightly attenuated in laboratory mice.

**FIGURE 9:**
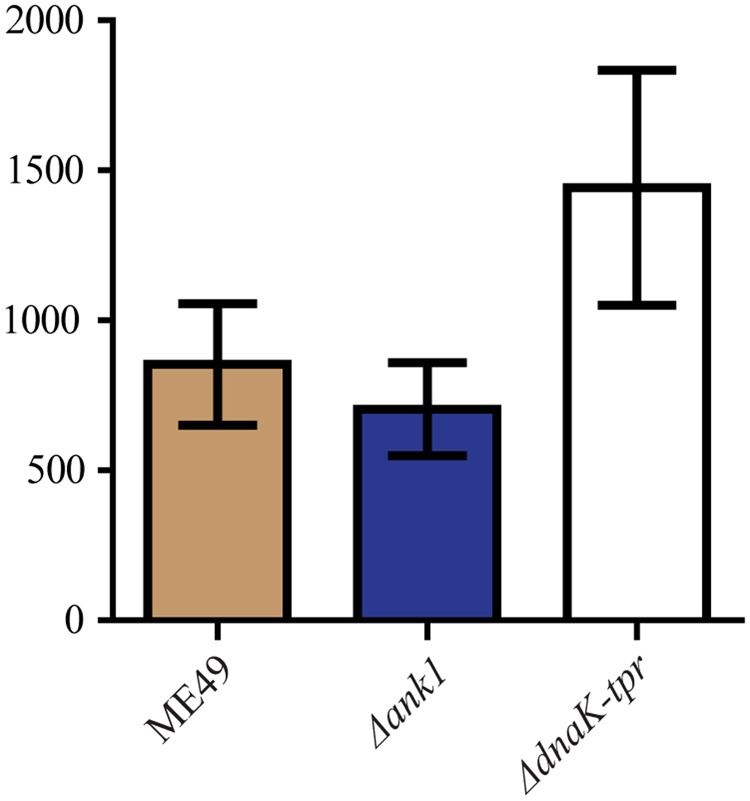
Cyst formation of the *ANK1* or *DnaK-TPR* deletion mutants *in vivo*. Purified tachyzoites from indicated strains were used to infect 10–12 weeks old Kunming mice (100 parasites/mouse) by intraperitoneal injection. Thirty days post infection, mice were sacrificed and the number of *Toxoplasma* cysts in each brain was determined by DBA-FITC staining and fluorescent microscopy. Mean ± SEM of cyst counting results from 8 mice for each strain.

Since the expression of both *ANK1* and *DnaK-TPR* are dramatically up-regulated in bradyzoites, we sought to determine whether they have any role in chronic infection establishment. To do that, 10–12 weeks old Kunming mice (genetically similar to ICR mice) were infected with 100 tachyzoites of each strain. Survival of mice was monitored daily and the number of cysts in the brain of mice that survived 30 days post infection was estimated. The use of 10–12 weeks old Kunming mice is because typical 4–6 weeks old ICR mice were susceptible to ME49 infection and no survivals could be obtained 30 days post infection. DBA-FITC staining and cyst counting suggested that there was no significant difference in brain cyst formation between the *ANK1* or *DnaK-TPR* knockout mutants and parental strain ME49 (**Figure 9**), indicating that neither gene directly contributes to chronic infection establishment.

## Discussion

Most of the *T. gondii* infection cases in animals and humans are chronic infection, where the parasites are encysted as bradyzoites. Under certain circumstances chronic infection can be re-activated and parasites can grow as tachyzoites to cause acute symptoms (Shen et al., 2016; Wohlfert et al., 2017). A large portion of human toxoplasmosis cases are derived from the reactivation of chronic infection, particularly in AIDS and organ transplantation patients (Pereira-Chioccola et al., 2009; Dubey et al., 2012; Meireles et al., 2015). In the wild, chronically infected animals are important sources of *T. gondii* transmission due to predation. Therefore bradyzoites play key roles in both pathogenesis and transmission of the parasites. However, the biological properties of bradyzoites are poorly understood, very little is known about how they are formed, how they persist and how they get re-activated. Great efforts using different strategies have been paid to compare the gene expression differences between tachyzoites and bradyzoites *in vitro* (Buchholz et al., 2011), or between acute and chronic infection stages *in vivo* (Pittman et al., 2014). Studies on these differentially regulated genes provided important insights into the biology of bradyzoite and stage conversion, such as the gene regulation by AP2 factors (White et al., 2014), cyst wall biogenesis (Tomita et al., 2013) and so on. In this study, we focused on two differentially expressed proteins, ANK1 and DnaK-TPR, that have novel TPR motifs involved in protein-protein interactions. Surprisingly, inactivation of either of the two proteins by CRISPR/CAS9 mediated gene knockout did not result in significant bradyzoite formation defect both *in vitro* and *in vivo*, although both mutants displayed modest virulence attenuation in mice. These results suggest that not all proteins that are predominantly expressed in bradyzoites are directly involved in bradyzoite formation, but may affect other aspects of the parasites.

One interesting observation from our study is the modest virulence attenuation of the Δ*ank1* and the Δ*dnaK-tpr* mutants. This is surprising because neither protein is significantly expressed in tachyzoites, therefore their inactivation was not expected to affect the acute virulence of the parasites. The exact mechanisms responsible for the reduced virulence of the mutants are currently unknown but possible explanations include: first, although both *ANK1* and *DnaK-TPR* are drastically up-regulated in bradyzoites, it is possible that they are expressed at very low levels in tachyzoites and the low levels of expression still contribute to the optimal virulence of the parasites. Particularly for *DnaK-TPR*, its transcript level is about 18% of that of *GAPDH* at the tachyzoite stage, therefore it is high likely that it is also expressed in tachyzoites, although at relatively low levels. Second, virulence attenuation may be due to indirect effects of gene deletions. Stage specific expression of genes is achieved through complex regulation mechanisms. Loss of the target genes may influence the local chromatin structure and/or the proper function of the regulation mechanisms, which in turn may affect the expression of other genes to alter the virulence properties. Further work is needed to test these possibilities.

Genes that are up or down regulated during bradyzoite conversion serve as good starting points to dissect the complex biology of bradyzoites. However, as the studies accumulate, it has become less clear whether these differentially regulated genes are actively contributing to bradyzoite conversion or their change in expression is just a byproduct of stage conversion. This is particularly true for bradyzoites induced *in vitro*. For example, the bradyzoite pseudokinase 1 (BPK1) is a component of the cyst wall upregulated in bradyzoites, however, inactivation of this gene did not affect pathogenesis or cyst formation *in vitro* (Buchholz et al., 2013). Its effect on cyst formation *in vivo* was also minimal, although it did affect the size and sensitivity of cysts to pepsin-acid (Buchholz et al., 2013). Similarly, deletion of the bradyzoite marker lactate dehydrogenase 2 (LDH2) did not affect bradyzoite transition *in vitro* (Abdelbaset et al., 2017) or cyst formation *in vivo* (Xia et al., to be published elsewhere). Disruption of another bradyzoite marker BAG1 also did not affect bradyzoite gene expression *in vitro* (Bohne et al., 1998; Zhang et al., 1999), nor did it affect cyst formation *in vivo* as reported in one study (Bohne et al., 1998). In this study when we knocked out either of the two TPR containing proteins (ANK1 and DnaK-TPR), no obvious phenotypes in bradyzoite conversion *in vitro* or cyst formation *in vivo* were observed, suggesting that they are not directly involved in chronic infection establishment. Currently it is not clear whether their increased expression in bradyzoites are just byproducts of parasite differentiation, or they actually play roles in other aspects of bradyzoites.

The two proteins we focused in this study were reported before as proteins whose expressions were significantly up-regulated during bradyzoite formation (Friesen et al., 2008; Ueno et al., 2011). But their exact roles were not known at the time of discovery. ANK1 contains an ankyrin repeat domain and a TPR domain. Similar to TPR, ankyrin repeats are also common protein motifs with 30–34 amino acid residues in length and function to mediate protein-protein interactions (Li et al., 2006). Like ANK1, DnaK-TPR also possesses two recognizable domains (DnaK/HSP70 family chaperone domain and TPR domain) that mediate protein-protein interactions. Because of the presence of these novel structural motifs, we initially thought that they might function as scaffolds to mediate protein complex formation during bradyzoite formation. In fact, DnaK-TPR was shown to interact with the p23 co-chaperone in a previous study (Ueno et al., 2011), which confirmed its ability to mediate protein interactions. Nonetheless, individual disruption of these two genes did not cause noticeable changes regarding bradyzoite differentiation and development. Possible reasons include: ANK1 and DnaK-TPR are not directly involved in cyst establishment, but may play roles in the structure integrity or physiological fitness of the cysts, which are not caught in our tests due to limited sensitivity. Alternatively, there are many other TPR containing proteins expressed in the parasites, at both tachyzoite and bradyzoite stages, which may functionally compensate the loss of ANK1 or DnaK-TPR, or ANK1 and DnaK-TPR themselves are functionally redundant. However, although both ANK1 and DnaK-TPR contain TPR motifs, the sequence similarity between them is very limited and TPRs are only small portions of these proteins. Therefore we think the likelihood that they are functionally redundant is low. Nonetheless, the possibilities that they functionally overlap with each other, or with other TPR containing proteins exist. Further work is required to examine these possibilities.

## Author Contributions

JY, LZ, and BS conceived and designed the study. JY, LZ, NX, and HD performed the experiments. BS, YZ, and JZ analyzed the data. BS, JY, and LZ wrote the paper.

## Conflict of Interest Statement

The authors declare that the research was conducted in the absence of any commercial or financial relationships that could be construed as a potential conflict of interest.

## Footnotes

1 https://blast.ncbi.nlm.nih.gov/Blast.cgi

2 http://myhits.isb-sib.ch/cgi-bin/motif_scan

3 https://toolkit.tuebingen.mpg.de/#/tools/tprpred
